# Newton-type algorithms for inverse optimization: weighted bottleneck Hamming distance and $\ell _\infty$-norm objectives

**DOI:** 10.1007/s11590-024-02183-0

**Published:** 2025-01-21

**Authors:** Kristóf Bérczi, Lydia Mirabel Mendoza-Cadena, Kitti Varga

**Affiliations:** MTA-ELTE Matroid Optimization Research Group, and HUN-REN–ELTE Egerváry Research Group, and Department of Operations Research, Eötvös Loránd University, Budapest, Hungary

**Keywords:** Combinatorial optimization, Algorithm, Bottleneck Hamming distance, Infinity norm, Inverse optimization

## Abstract

In inverse optimization problems, we are given a feasible solution to an underlying optimization problem, and the goal is to modify the problem parameters so that the given input solution becomes optimal. In the minimum-cost setting, the underlying optimization problem is endowed with a linear cost function, and the goal is to modify the costs by a small deviation vector so that the input solution becomes optimal. The difference between the new and the original cost functions can be measured in several ways. In this paper, we focus on two objectives: the weighted bottleneck Hamming distance and the weighted $\ell _{\infty }$-norm. We consider a general model in which the coordinates of the deviation vector are required to fall within given lower and upper bounds. For the weighted bottleneck Hamming distance objective, we present a simple, purely combinatorial algorithm that determines an optimal deviation vector in strongly polynomial time. For the weighted $\ell _{\infty }$-norm objective, we give a min-max characterization for the optimal solution, and provide a pseudo-polynomial algorithm for finding an optimal deviation vector that runs in strongly polynomial time in the case of unit weights. For both objectives, we assume that an algorithm with the same time complexity for solving the underlying combinatorial optimization problem is available.

## Introduction

In a classical optimization problem, we are given a set of feasible solutions together with a linear cost function, and the goal is to find a feasible solution that minimizes or maximizes the cost. In contrast, in an inverse optimization problem we are also given a fixed feasible solution, and the goal is to modify the costs ‘as little as possible’ so that the input solution becomes optimal. There are various ways to measure the deviation of the new cost function from the original one, and, as one would expect, the choice of the objective greatly affects the complexity of the problem. The probably most standard objective functions are the $\ell _1$- and $\ell _{\infty }$-norms and the different Hamming distances. Minimizing the $\ell _1$-norm of the deviation vector means that the overall change in the costs is small, while minimizing the $\ell _{\infty }$-norm results in a new cost function that is close to the original one coordinate-wise. Minimizing the Hamming distance means that the extent of modification is irrelevant coordinate-wise, but the aim is to make a modification in a few coordinates only: when considering the sum-type and the bottleneck Hamming distances, the goal is to minimize the sum and the maximum of the weights of those coordinates where a modification is made, respectively. In order to avoid confusion, we refer to the solutions of the inverse optimization problem and those of the underlying combinatorial optimization problem as *feasible deviation vectors* and *solutions*, respectively.

Inverse problems have long been the focus of research due to their wide applicability in both theory and practice, and were first studied by geophysical scientists [32]. One of the early applications of inverse optimization in geophysics was to predict the movements of earthquakes [29, 37]. The roots of the interest in inverse optimization within the mathematical programming community go back to the work of Burton and Toint [8] who studied the inverse shortest paths problem, that is, the problem of recovering the edge costs given some information about the shortest paths in the graph. Since their pioneering work, countless of applications and extensions emerged; we refer the interested reader to [10, 12, 21] for surveys. Besides theoretical results, inverse optimization problems also found numerous applications. For example, Chan et al. [9] considered the pathway concordance problem to develop a data-driven concordance metric for measuring how much patients’ care follows the recommendations set up by medical experts, with the aim of optimizing survival, cost, and waiting times. In another work, Chen, Chen, and Langevin [11] studied a capacitated vehicle routing problem that appeared as part of a collaboration with an e-commerce company.

To motivate the objective functions considered, let us mention that $\ell _1$-, $\ell _\infty$-norm and Hamming distance objectives play a crucial role, e.g., in designing communication networks. In such a network, every edge is assigned a bandwidth, indicating the maximum amount of data that can be transferred along that edge. The communication between nodes is performed through the edges of a fixed spanning tree, whose bandwidth is the minimum bandwidth of its edges. A basic problem is to make the currently used spanning tree have maximum bandwidth by modifying the bandwidth of some edges. Here, one can add natural restrictions on the modifications. For example, maybe we can only increase the bandwidths of the edges, or we might have only limited amount of resources to do so. Minimizing the total amount of bandwidth change can be modelled as minimizing the $\ell _1$-norm. If our goal is to keep the new bandwidths as close to the original ones as possible, then the problem can be formulated using the $\ell _{\infty }$-norm. If the time of executing the modifications is considered and it is independent of the extent of the modification in bandwidth, then different variants of the Hamming distance are helpful: if the modifications are carried out consecutively then the sum-type Hamming distance, while if the modifications are made simultaneously then the bottleneck Hamming distance describes the objective. Similar applications are described by Faragó, Szentesi, and Szviatovszki [15].

The present work is part of a series of papers aiming to provide min-max characterizations and simple algorithms for inverse optimization problems under various objectives. In [6], we established a general framework based on a Newton-type approach for finding an optimal deviation vector, deriving an algorithm specifically for the weighted span objective to identify a ‘balanced’ or ‘fair’ deviation vector. Here, we develop similar algorithms for two additional objective classes: the weighted bottleneck Hamming distance and weighted $\ell _{\infty }$-norm. We adopt the notation, definitions, and terminology from this prior work.

Our algorithms are termed “Newton-type” in line with the convention set by Zhang and Liu [41]; see also Heuberger [21]. Recall that Newton’s method seeks to find a root $x^*$ of a function *f*. In our setting, we are given an input solution $F^*$, and we want to change the cost function so that $F^*$ becomes optimal. Thus we analyze the current optimal solution *F*, analogous to the current point *x* in Newton’s method. If the cost of *F* matches that of $F^*$, we stop; similarly, Newton’s method terminates if *x* is a root of *f*. If not, we adjust the costs to eliminate *F*, that is, to make $F^*$ and *F* have equal costs; in Newton’s method, the update involves moving from *x* to the root of the first-order approximation of *f*. Further explanation of the naming convention can be found in Remark 14.

### Previous work

The *inverse maximum-weight perfect matching problem* was studied under the weighted bottleneck Hamming distance with bounds given on the modification by Liu and Yao [26] who provided a Newton-type algorithm. Later Tayyebi [33] suggested a faster algorithm that relies on binary search. Liu and Zhang [27] presented a binary search algorithm and a Newton-type algorithm for the *inverse maximum-weight matching problem* under the $\ell _{\infty }$-norm. Ahuja and Orlin [3] solved the *inverse minimum-cut problem* under the $\ell _{\infty }$-norm with non-negativity constrains using binary search. Duin and Volgenant [14] proposed a greedy algorithm for the *inverse minimum spanning tree problem* under the weighted bottleneck Hamming distance. The *inverse linear assignment problem* under the weighted bottleneck Hamming distance was studied by Duin and Volgenant [14] who proposed a greedy algorithm. The bounded case was solved by Tayyebi [33] using a binary search method. Wang, Yang, and Wu [36] also studied the inverse assignment problem but under the $\ell _{\infty }$-norm with bounds given on the modification, and provided a binary search algorithm for solving it. Aman, Hassanpour and Tayyebi [4] provided an algorithm for the *inverse maximum-cost base problem* in matroids under the weighted bottleneck Hamming distance with bounds given on the modification. It is worth emphasizing that the algorithms of the current paper are applicable for all the above mentioned problems.

The *inverse maximum-cost base problem*, where the goal is to modify the costs so that each input element is contained in a maximum-cost basis, was considered by Li, Zhang, and Lai [25] under the $\ell _{\infty }$-norm objective when the costs can only be decreased, and by Zhang, Li, Lai, and Du [42] under any non-decreasing norm when the costs can only be increased. Using linear programming (LP) techniques, Ahuja and Orlin [2] proved that if an optimization problem can be modeled as an LP, then the same holds for the underlying *inverse linear programming problem* under $\ell _1$- or $\ell _\infty$-norm objectives. Furthermore, if the optimization problem is polynomially solvable for linear cost functions, then the inverse counterparts with $\ell _1$- and $\ell _{\infty }$-norms are also polynomially solvable. When the LP fulfils certain requirements, the inverse linear programming problem under the $\ell _{\infty }$-norm was solved by Zhang and Liu [40] using an LP-based algorithm, and by Zhang and Liu [41] using a Newton-type algorithm. Under the weighted bottleneck Hamming distance with bounds given on the modification, Tayyebi and Aman [34] proposed a binary search algorithm. Xiaoguang [38] studied the *inverse optimization of submodular functions* on digraphs under the $\ell _{\infty }$-norm with bounds given on the modification, and the author presented an LP-based algorithm for this problem. The *inverse discrete p-median problem* with bounds given on the modification, was considered by Burkard, Pleschiutschnig and Zhang [7] and they proposed a greedy algorithm for it. When $p=1$ and the underlying graph is a tree, this problem was solved by Guan and Zhang under the weighted bottleneck Hamming distance using a binary search algorithm [20] and under the weighted $\ell _{\infty }$-norm using a greedy algorithm [19, 20]. Qian, Guan, Jia, Zhang, and Pardalos [30] proposed a binary search algorithm for the *inverse vertex quickest 1-center problem* on a tree under the weighted $\ell _{\infty }$-norm with bounds given on the modification. Jiang, Liu, and Peng [22] provided a combinatorial algorithm for the *inverse minimum-value flow problem* when there are no parallel arcs in the network with bounds given on the modification under the weighted bottleneck Hamming distance. The *inverse generalized minimum-cost flow problem* under the weighted bottleneck Hamming distance with bounds given on the modification was solved using an LP-based algorithm by Karimi, Aman, and Dolati [23] assuming we have access to dual solutions. The inverse generalized minimum-cost flow problem with bounds given on the modification was also studied by Tayyebi and Deaconu [35] who proposed combinatorial algorithms for both the weighted bottleneck Hamming distance and the weighted $\ell _{\infty }$-norm when there are no parallel arcs in the network. Ahmadian, Bhaskar, Sanità, and Swamy [1] studied integral inverse optimization problems from an approximation point of view. They obtained tight or nearly-tight approximation guarantees for various inverse optimization problems, and some of their results apply for $\ell _{\infty }$-norm as well. The paper [5] introduced inverse optimization problems with multiple cost functions, and studied the inverse minimum-cost *s*-*t* path, *r*-arborescence, and bipartite perfect matching problems under the $\ell _1$-norm.

Significant amount of work has been made on inverse optimization problems when the underlying optimization problem uses a non-linear cost function. Mohaghegh and Baroughi Bonab [28] studied the *inverse min-max spanning r-arborescence problem* under the weighted Hamming distance. Guan, He, Pardalos, and Zhang [18] considered the *inverse max+sum spanning tree problem* under the weighted Hamming distance. Dong, Li, and Yang [13] addressed the *partial inverse min-max spanning tree problem* under the weighted Hamming distance. Yang and Zhang [39] solved the *inverse min-max spanning tree* and the *inverse maximum-capacity path problem* under the $\ell _{\infty }$-norm. Lasserre [24] considered the *inverse polynomial optimization problem* under the $\ell _{\infty }$-norm.

Most papers on inverse optimization consider algorithmic aspects, and so they do not provide a min-max characterization for the optimum value in question. A min-max characterization can be used to easily validate the optimality of a feasible solution. Frank and Hajdu [16] gave a min-max formula for the minimum modification of the cost function in the inverse arborescence problem, together with a simpler combinatorial algorithm than previous ones. Recently, Frank and Murota [17] developed a general min-max formula for the minimum of an integer-valued separable discrete convex function, where the minimum is taken over the set of integral elements of a box total dual integral polyhedron. Their approach covers and even extends a wide class of inverse combinatorial optimization problems. Nevertheless, our problems do not fit in the box-TDI framework as neither the bottleneck Hamming distance nor the $\ell _\infty$-norm is separable convex.

### Problem definitions

To make the paper self-contained, we repeat the basic notation and definitions as summarized in [6].

We denote the sets of *real* and *positive real* numbers by ${\mathbb {R}}$ and ${\mathbb {R}}_+$, respectively. For a positive integer *k*, we use $[k]:=\{1,\dots ,k\}$. Let *S* be a ground set of size *n*. Given subsets $X,Y\subseteq S$, the *symmetric difference* of *X* and *Y* is denoted by $X\triangle Y:=(X{\setminus } Y)\cup (Y{\setminus } X)$. By convention, we define $\min \emptyset =+\infty$ and $\max \emptyset =-\infty$.

Let *S* be a finite ground set, ${\mathcal {F}}\subseteq 2^S$ be a collection of *feasible solutions* for an underlying optimization problem, $F^* \in {\mathcal {F}}$ be an *input solution*, $c \in {\mathbb {R}}^S$ be *costs*, $w\in {\mathbb {R}}_+^S$ be positive *weights*, and $\ell :S\rightarrow {\mathbb {R}}\cup \{-\infty \}$ and $u :S\rightarrow {\mathbb {R}}\cup \{+\infty \}$ be *lower and upper bounds*, respectively, such that $\ell \le u$. For the costs $c \in {\mathbb {R}}^S$, the cost of a set $F \in {\mathcal {F}}$ is defined as $c(F) :=\sum _{s \in F} c(s)$ where *c*(*s*) represents the *s*-th component of *c*. For the weights $w\in {\mathbb {R}}_+^S$, the total sum of its values over *X* is denoted by $w(X):=\sum _{s \in X} w(s)$, where the sum over the empty set is always considered to be 0 and where *w*(*s*) represents the *s*-th component of *w*. Furthermore, we define $\frac{1}{w}(X):=\sum _{s \in X} \frac{1}{w(s)}$, and set $\Vert w\Vert _{\text {-}1}:=\frac{1}{w}(S)$. When the weights are rational numbers, the values can be re-scaled as to satisfy 1/*w*(*s*) being an integer for each $s\in S$. Throughout the paper, we assume that *w* is given in such a form without explicitly mentioning it, implying that $\frac{1}{w}(X)$ is a non-negative integer for every $X\subseteq S$.

We assume that an *oracle* ${\mathcal {O}}$ is also available that determines an optimal solution of the underlying optimization problem $(S, {\mathcal {F}}, c')$ for any costs $c'\in {\mathbb {R}}^S$.

In the *constrained minimum-cost inverse optimization problem under the weighted bottleneck Hamming distance objective*, denoted by $\big ( S, {\mathcal {F}}, F^*, c, \ell , u, \textrm{H}_{\infty ,w}(\cdot ) \big )$, we seek a *deviation vector* $p \in {\mathbb {R}}^S$ such that

- $F^*$ is a minimum-cost member of ${\mathcal {F}}$ with respect to $c-p$,
- *p* is within the bounds $\ell \le p\le u$, and
- $$\textrm{H}_{\infty ,w}(p) :=\max \left\{ w(s)\mid s \in S, \, p(s) \ne 0 \right\}$$ is minimized.

In the *constrained minimum-cost inverse optimization problem under weighted* $\ell _\infty$*-norm objective*, denoted by $( S, {\mathcal {F}}, F^*, c, \ell , u, \Vert \cdot \Vert _{\infty , w})$, condition (c) modifies to

(c’): $$\Vert p\Vert _{\infty , w} :=\max \left\{ w(s) \cdot |p(s)| \bigm | s \in S \right\}$$ is minimized.

Due to the lower and upper bounds $\ell$ and *u*, it might happen that there exists no deviation vector *p* satisfying the requirements. A deviation vector is called *feasible* if it satisfies conditions (a) and (b), and *optimal* if in addition it attains the minimum in (c) or (c’). We denote the problems by $\big ( S, {\mathcal {F}}, F^*, c, -\infty , +\infty , \textrm{H}_{\infty ,w}(\cdot ) \big )$ and $( S, {\mathcal {F}}, F^*, c, -\infty , +\infty ,\Vert \cdot \Vert _{\infty , w} )$ when no bounds are given on the coordinates of *p* at all, and call these problems *unconstrained*.

### Our results

 Our main results are simple, purely combinatorial algorithms that efficiently solve the above, general problems. The emphasis is not primarily on runtime but on introducing a flexible and versatile framework that is capable of handling general inverse optimization problems where feasible solutions in ${\mathcal {F}}$ might have different sizes, and lower and upper bounds might be given on the coordinates of the deviation vector *p*. Note that since *S* is finite and ${\mathcal {F}}\subseteq 2^S$, our framework covers purely combinatorial problems.

The proposed algorithms differ from conventional inverse optimization approaches, such as binary search and LP-based methods. Instead, we suggest an algorithm that iteratively updates the costs, resembling the approach of Zhang and Liu [41] for the $\ell _{\infty }$-norm objective. They showed that if an inverse optimization problem can be reformulated as a certain maximization problem using dominant sets, then Radzik’s method [31] yields a strongly polynomial algorithm to find an optimal solution. In contrast, our algorithms are applicable to a broader range of inverse optimization problems, as they do not require a reformulation. Rather, we only rely on the fact that the underlying optimization problem is solvable. The reformulation proposed by Zhang and Liu [41] is generally $\textsf{NP}$-hard, except in special cases such as the minimum spanning tree, minimum-cost flow (e.g., shortest path, assignment), maximum perfect matching, maximum intersection of two matroids (e.g., maximum weighted directed tree, minimum spanning tree with partition constraints), the reverse bottleneck expansion problem, and fractional binary optimization. Furthermore, we consider the constrained setting in which the coordinates of the deviation vector are ought to fall within given lower and upper bounds, and hence the costs have to be updated carefully. For these reasons, Radzik’s method cannot be applied to get a strongly polynomial algorithm.

For the weighted bottleneck Hamming distance objective, we present two algorithms: one that aligns with our series of Newton-type algorithms, and the second one that is faster. The first algorithm is a Newton-type algorithm that makes *O*(*n*) calls to the oracle ${\mathcal {O}}$. In particular, the algorithm runs in strongly polynomial time, assuming that a strongly polynomial algorithm for the underlying optimization problem is available. The second algorithm uses binary search and makes $O(\log n)$ calls to the oracle ${\mathcal {O}}$. For the weighted $\ell _{\infty }$-norm objective, we give an algorithm for finding an optimal deviation vector that makes $O(n\cdot \Vert w\Vert _{\text {-}1})$ calls to the oracle ${\mathcal {O}}$. In particular, the algorithm runs in strongly polynomial time for unit weights if the oracle ${\mathcal {O}}$ for the underlying optimization problem can be realized by a strongly polynomial algorithm. Furthermore, we provide a min-max characterization for the minimum size of an optimal deviation vector in the unconstrained setting, i.e. when $\ell \equiv -\infty$ and $u \equiv +\infty$.

A high-level description of our Newton-type algorithm is given by the following scheme.

**Step 1.**: Choose $p_0$ minimizing the objective such that $\ell \le p_0\le u$, set $c_0:=c-p_0$ and $i :=0$.
**Step 2.**: Let $F_i$ be an optimal solution of the underlying optimization problem with respect to $c_i$.
**Step 3.**: If $c_i(F^*)=c_i(F_i)$, then $p_i$ is an optimal deviation vector and stop. Otherwise, find $p_{i+1}$ satisfying $\ell \le p_{i+1}\le u$ and $(c-p_{i+1})(F^*)=(c-p_{i+1})(F_i)$, and minimizing the objective. If no such $p_{i+1}$ exists, then the problem is infeasible and stop. Otherwise set $c_{i+1} :=c - p_{i+1}$ and $i \leftarrow i+1$, and go back to Step 2.

The rest of the paper is organized as follows. Section 2 presents two strongly polynomial algorithms for the weighted bottleneck Hamming distance objective: one that fits in our framework, and another one that relies on binary search. The weighted $\ell _{\infty }$-norm objective is discussed in Sect. 3, where we first give an algorithm for the constrained setting, and then we provide a min-max characterization for the weighted $\ell _{\infty }$-norm of an optimal deviation vector in the unconstrained setting.

## Weighted bottleneck Hamming distance objective

As a warm-up, we first consider the problem of minimizing the weighted bottleneck Hamming distance. In Sect. 2.1, we show that there exists an optimal deviation vector having a restricted structure. We characterize the feasibility of the problem in Sect. 2.2. A Newton-type algorithm is presented in Sect. 2.3, while a binary search algorithm is given in Sect. 2.4.

### Optimal deviation vectors

Consider the constrained minimum-cost inverse optimization problem under the weighted bottleneck Hamming distance objective $\big ( S, {\mathcal {F}}, F^*, c, \ell , u, \textrm{H}_{\infty ,w}(\cdot ) \big )$, where ${w\in {\mathbb {R}}^S_+}$ are positive weights. For ease of discussion, we define

$$\begin{aligned} m :=\max \left\{ \max _{s \in F^*} \big \{ \ell (s) \big \}, \max _{s \in S \setminus F^*} \! \big \{ \! - \! u(s) \big \}, ~ \max _{F \in {\mathcal {F}}} \left( c(F^*) - c(F) - \!\! \sum _{\begin{array}{c} s \in F^*\setminus F \\ u(s) < 0 \end{array}} \! u(s) + \! \sum _{\begin{array}{c} s \in F\setminus F^* \\ \ell (s) > 0 \end{array}} \! \ell (s) \right) \! \right\} . \end{aligned}$$

The intuition for the definition of *m* is the following. Assume that $F\in {\mathcal {F}}$ has smaller cost than $F^*$ and that *s* is an element in $F^*\mathbin {\triangle } F$ whose cost can be changed arbitrarily, that is, $\ell (s)=-\infty$ and $u(s)=+\infty$. Then, the value of *m* is large enough to make the cost of $F^*$ less than the one of *F* by setting *p*(*s*) to *m* if $s\in F^*\setminus F$ and to $-m$ if $s\in F\setminus F^*$.

Recall that ${\mathcal {O}}$ denotes an algorithm that determines an optimal solution of the underlying optimization problem $(S, {\mathcal {F}}, c')$ for any costs $c' \in {\mathbb {R}}^S$. Observe that if ${\mathcal {O}}$ runs in strongly polynomial time, then the value of *m* can be determined in strongly polynomial time. To see this, define the new costs ${\widetilde{c}} \in {\mathbb {R}}^S$ by setting ${\widetilde{c}}(s):=c(s)-u(s)$ if $u(s) < 0$ and $s\in F^*$, and by ${\widetilde{c}}(s) :=c(s)-\ell (s)$ if $\ell (s) > 0$ and $s\in S\setminus F^*$, and by ${\widetilde{c}}(s) :=c(s)$ otherwise. Let ${\widetilde{C}}$ denote the cost of an optimal solution of $(S, {\mathcal {F}}, {\widetilde{c}})$ that can be determined using ${\mathcal {O}}$. Then ${\widetilde{c}}(F^*) - {\widetilde{C}}$ gives the third item in the definition of *m*, while the other two items are easy to determine.

For any $\delta \ge 0$, let $p_{[\delta |\ell , u|w]}:S\rightarrow {\mathbb {R}}$ be defined as

$$\begin{aligned} p_{[\delta |\ell , u|w]}(s):={\left\{ \begin{array}{ll} u(s) & \text {if }s \in F^*,\ u(s) \ne + \infty \text { and }w(s) \le \delta , \\ m & \text {if }s \in F^*,\ u(s) = + \infty \text { and }w(s) \le \delta , \\ \ell (s) & \text {if }s \in S\setminus F^*,\ \ell (s) \ne - \infty \text { and }w(s) \le \delta , \\ -m & \text {if }s \in S\setminus F^*,\ \ell (s) = - \infty \text { and }w(s) \le \delta , \\ 0 & \text {otherwise}. \end{array}\right. } \end{aligned}$$

The following lemma shows that there exists an optimal deviation vector of special form.

#### Lemma 1

Let $\big ( S, {\mathcal {F}}, F^*, c, \ell , u, \textrm{H}_{\infty , w}(\cdot ) \big )$ be a feasible minimum-cost inverse optimization problem and let $p_{{\textsc {opt}}}$ be an optimal deviation vector. Then, $p_{[\delta |\ell , u|w]}$ is also an optimal deviation vector, where $\delta :=\textrm{H}_{\infty , w}(p_{{\textsc {opt}}})$.

#### Proof

The lower and upper bounds $\ell \le p_{[\delta |\ell ,u|w]}\le u$ hold by definition, and hence (b) is satisfied.

Now we show that (a) holds. Let $F \in {\mathcal {F}}$ be an arbitrary solution. Then

$$\begin{aligned}&(c - p_{[\delta |\ell , u|w]})(F^*) - (c - p_{[\delta |\ell , u|w]})(F)\\&\quad = \left( c(F^*) - \sum _{s \in F^*} p_{[\delta |\ell , u|w]}(s) \right) - \left( c(F) - \sum _{s \in F} p_{[\delta |\ell , u|w]}(s) \right) \\&\quad = c(F^*) - c(F) - \sum _{s \in F^*\setminus F} p_{[\delta |\ell , u|w]}(s) + \sum _{s \in F\setminus F^*} p_{[\delta |\ell , u|w]}(s) \\&\quad = c(F^*) - c(F) - \sum _{\begin{array}{c} s \in F^* \setminus F \\ u(s) \ne + \infty \\ w(s) \le \delta \end{array}} u(s) - \sum _{\begin{array}{c} s \in F^*\setminus F \\ u(s) = + \infty \\ w(s) \le \delta \end{array}} m + \sum _{\begin{array}{c} s \in F \setminus F^* \\ \ell (s) \ne - \infty \\ w(s) \le \delta \end{array}} \ell (s) + \sum _{\begin{array}{c} s \in F \setminus F^* \\ \ell (s) = - \infty \\ w(s) \le \delta \end{array}} (-m) . \end{aligned}$$

If $$\big \{ s \in F^*{\setminus } F \bigm | u(s) = + \infty ,\ w(s)\le \delta \big \} \cup \big \{ s \in F {\setminus } F^* \bigm | \ell (s) = - \infty ,\ w(s)\le \delta \big \} = \emptyset$$, then

$$\begin{aligned}&(c - p_{[\delta |\ell , u|w]})(F^*) - (c - p_{[\delta |\ell , u|w]})(F)\\&\quad = c(F^*) - c(F) - \sum _{\begin{array}{c} s \in F^*\setminus F \\ u(s) \ne + \infty \\ w(s) \le \delta \end{array}} u(s) + \sum _{\begin{array}{c} s \in F \setminus F^* \\ \ell (s) \ne - \infty \\ w(s) \le \delta \end{array}} \ell (s) \\&\quad \le c(F^*) - c(F) - \sum _{\begin{array}{c} s \in F^*\setminus F \\ u(s) \ne + \infty \\ w(s) \le \delta \end{array}} p_{{\textsc {opt}}}(s) + \sum _{\begin{array}{c} s \in F \setminus F^* \\ \ell (s) \ne - \infty \\ w(s) \le \delta \end{array}} p_{{\textsc {opt}}}(s)\\&\quad = (c-p_{{\textsc {opt}}})(F^*) - (c-p_{{\textsc {opt}}})(F)\\&\quad \le 0. \end{aligned}$$

Otherwise, $\big \{ s \in F^*{\setminus } F \bigm | u(s) = + \infty ,\ w(s)\le \delta \big \} \cup \big \{ s \in F {\setminus } F^* \bigm | \ell (s) = - \infty ,\ w(s)\le \delta \big \} \ne \emptyset$. Note that, by the feasibility of $p_{{\textsc {opt}}}$ and by the definition of $\delta$, we have $\ell (s) \le 0 \le u(s)$ whenever $w(s) > \delta$. Thus, we obtain

$$\begin{aligned}&(c - p_{[\delta |\ell , u|w]})(F^*) - (c - p_{[\delta |\ell , u|w]})(F)\\&\quad = c(F^*) - c(F) - \sum _{\begin{array}{c} s \in F^*\setminus F \\ u(s) \ne + \infty \\ w(s) \le \delta \end{array}} u(s) - \sum _{\begin{array}{c} s \in F^*\setminus F \\ u(s) = + \infty \\ w(s) \le \delta \end{array}} m + \sum _{\begin{array}{c} s \in F \setminus F^* \\ \ell (s) \ne - \infty \\ w(s) \le \delta \end{array}} \ell (s) + \sum _{\begin{array}{c} s \in F \setminus F^* \\ \ell (s) = - \infty \\ w(s) \le \delta \end{array}} (-m) \\&\quad \le c(F^*) - c(F) - \sum _{\begin{array}{c} s \in F^*\setminus F \\ u(s) \ne + \infty \\ w(s) \le \delta \end{array}} u(s) + \sum _{\begin{array}{c} s \in F \setminus F^* \\ \ell (s) \ne - \infty \\ w(s) \le \delta \end{array}} \ell (s) - 1 \cdot m \\&\quad \le c(F^*) - c(F) - \sum _{\begin{array}{c} s \in F^*\setminus F \\ u(s)< 0 \\ w(s) \le \delta \end{array}} u(s) + \sum _{\begin{array}{c} s \in F \setminus F^* \\ \ell (s)> 0 \\ w(s) \le \delta \end{array}} \ell (s) - m \\&\quad = c(F^*) - c(F) - \sum _{\begin{array}{c} s \in F^*\setminus F \\ u(s) < 0 \end{array}} u(s) + \sum _{\begin{array}{c} s \in F \setminus F^* \\ \ell (s) > 0 \end{array}} \ell (s) - m\\&\quad \le 0, \end{aligned}$$

where the last inequality holds by the definition of *m*. Therefore, (a) is indeed satisfied.

Finally, to see (c), observe that $\textrm{H}_{\infty , w}(p_{[\delta |\ell , u|w]}) \le \delta = \textrm{H}_{\infty , w}(p_{{\textsc {opt}}})$, and hence $p_{[\delta |\ell , u|w]}$ is also optimal. $\square$

By Lemma 1, it suffices to look for an optimal deviation vector among vectors of special form. It turns out that the value of $\delta$ can be chosen from the values of the weights *w*.

#### Lemma 2

Let $\big ( S, {\mathcal {F}}, F^*, c, \ell , u, \textrm{H}_{\infty , w}(\cdot ) \big )$ be a feasible minimum-cost inverse optimization problem and let $\delta \ge 0$ be such that $p_{[\delta |\ell , u|w]}$ is a feasible deviation vector. Then, the following hold.

(i) There exists $s \in S$ with $w(s) \le \delta$ for which $p_{[w(s)|\ell , u|w]}$ is also a feasible deviation vector, or $p_{[0|\ell , u|w]}$ is also a feasible deviation vector.
(ii) For any $\delta ' \ge \delta$, the deviation vector $p_{[\delta '|\ell , u|w]}$ is also feasible.

#### Proof

To see (i), consider the set $\{ w(s') \mid s' \in S, \, w(s') \le \delta \}$. If this set is empty, then by definition $p_{[\delta |\ell , u|w]} = p_{[0|\ell , u|w]}$. If this set is non-empty, then let $s \in S$ be such an element for which $w(s) = \max \{ w(s') \mid s' \in S, \, w(s') \le \delta \}$ holds. Then, by definition we have $p_{[\delta |\ell , u|w]} = p_{[w(s)|\ell , u|w]}$.

For (ii), let $F \in {\mathcal {F}}$ be arbitrary. Note that, by the feasibility of $p_{[\delta |\ell , u|w]}$, we have $\ell (s) \le 0 \le u(s)$ whenever $w(s) > \delta$. Then

$$\begin{aligned}&(c - p_{[\delta '|\ell , u|w]})(F^*) - (c - p_{[\delta '|\ell , u|w]})(F)\\&\quad = c(F^*) - c(F) - \sum _{\begin{array}{c} s \in F^*\setminus F \\ u(s) \ne + \infty \\ w(s) \le \delta ' \end{array}} u(s) - \sum _{\begin{array}{c} s \in F^*\setminus F \\ u(s) = + \infty \\ w(s) \le \delta ' \end{array}} m + \sum _{\begin{array}{c} s \in F \setminus F^* \\ \ell (s) \ne - \infty \\ w(s) \le \delta ' \end{array}} \ell (s) + \sum _{\begin{array}{c} s \in F \setminus F^* \\ \ell (s) = - \infty \\ w(s) \le \delta ' \end{array}} (-m) \\&\quad \le c(F^*) - c(F) - \sum _{\begin{array}{c} s \in F^*\setminus F \\ u(s) \ne + \infty \\ w(s) \le \delta \end{array}} u(s) - \sum _{\begin{array}{c} s \in F^*\setminus F \\ u(s) = + \infty \\ w(s) \le \delta \end{array}} m + \sum _{\begin{array}{c} s \in F \setminus F^* \\ \ell (s) \ne - \infty \\ w(s) \le \delta \end{array}} \ell (s) + \sum _{\begin{array}{c} s \in F \setminus F^* \\ \ell (s) = - \infty \\ w(s) \le \delta \end{array}} (-m) \\&\quad = (c - p_{[\delta |\ell , u|w]})(F^*) - (c - p_{[\delta |\ell , u|w]})(F)\\&\quad \le 0, \end{aligned}$$

concluding the proof of the lemma. $\square$

### Characterizing feasibility

We give a necessary and sufficient condition for the feasibility of the minimum-cost inverse optimization problem $\big ( S, {\mathcal {F}}, F^*, c, \ell ,u, \textrm{H}_{\infty ,w}(\cdot ) \big )$.

#### Lemma 3

The minimum-cost inverse optimization problem $\big ( S, {\mathcal {F}}, F^*, c, \ell , u, \textrm{H}_{\infty , w}(\cdot ) \big )$ is feasible if and only if $p_{[w_{\max }|\ell , u|w]}$ is a feasible deviation vector, where $w_{\max } \hspace{-2.25pt} :=\hspace{-1.75pt} \max \! \left\{ \hspace{-0.5pt} w(s) \hspace{-2.25pt} \bigm | \hspace{-1.5pt} s \! \in \! S \hspace{-0.15pt} \right\}$.

#### Proof

Clearly, if $p_{[w_{\max }|\ell , u|w]}$ is feasible, then so is the problem.

To see the other direction, assume that the problem is feasible. Let *p* be an optimal deviation vector and set $\delta :=\textrm{H}_{\infty , w}(p)$. Then, by Lemma 1, the deviation vector $p_{[\delta |\ell , u|w]}$ is also optimal. By Lemma 2, this implies the feasibility of $p_{[w_{\max }|\ell , u|w]}$. $\square$

### Newton-type algorithm

We turn to the description of the algorithm and its analysis. The high-level idea is as described in the introduction. In each iteration, we determine an optimal solution $F\in {\mathcal {F}}$ using the oracle ${\mathcal {O}}$ as a black box. If the cost of *F* equals that of $F^*$, then we stop. Otherwise, we modify the costs in such a way that *F* is “eliminated”, that is, *F* and $F^*$ share the same cost with respect to the modified costs – hence the name *Newton-type*. The algorithm is presented as Algorithm 1.

**Algorithm 1:**
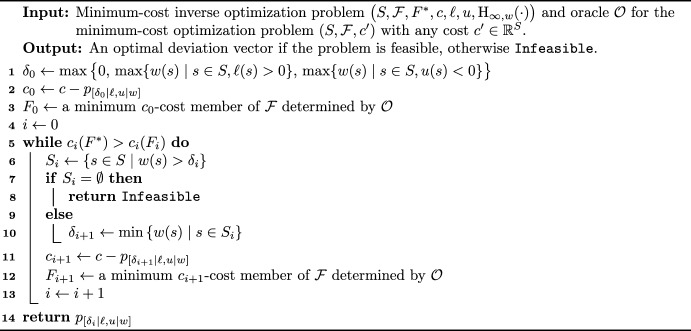
Algorithm for the constrained minimum-cost inverse optimization problem under the weighted bottleneck Hamming distance objective

It remains to prove the correctness and the running time of the algorithm.

#### Theorem 4

Algorithm 1 determines an optimal deviation vector, if exists, for the minimum-cost inverse optimization problem $\big ( S, {\mathcal {F}}, F^*, c, \ell , u, \textrm{H}_{\infty , w}(\cdot ) \big )$ using *O*(*n*) calls to the oracle ${\mathcal {O}}$.

#### Proof

We discuss the time complexity and the correctness of the algorithm separately.

*Time complexity.* We show that the algorithm terminates after at most *n* iterations of the while loop. To see this, observe that if $F^*$ is not a minimum $c_i$-cost member of ${\mathcal {F}}$ for some *i*, then either $S_{i+1} \subsetneq S_i$ by the definition of $\delta _{i+1}$, or the algorithm declares the problem to be infeasible. As the size of the set $S_i$ can decrease at most $|S|=n$ times, the statement follows.

*Correctness.* By the above, the algorithm terminates after a finite number of iterations. Observe that if the algorithm returns Infeasible, then it correctly recognizes the problem to be infeasible by Lemma 3.

Assume now that the algorithm terminates with returning a deviation vector $p_{[\delta _i|\ell , u|w]}$ whose feasibility follows from the fact that the while loop ended. If $F^*$ is a minimum $c_0$-cost member of ${\mathcal {F}}$, then we are clearly done. Otherwise, there exists an index *q* such that $F^*$ is a minimum $c_{q+1}$-cost member of ${\mathcal {F}}$. Suppose to the contrary that $p_{[\delta _{q+1}|\ell , u|w]}$ is not optimal. Since the problem is feasible, by Lemma 1, there exists $\delta <\delta _{q+1}$ such that the deviation vector $p_{[\delta |\ell , u|w]}$ is optimal. Note that $p_{[\delta _q|\ell , u|w]}$ is not a feasible deviation vector since $(c - p_{[\delta _q|\ell , u|w]})(F^*) > (c - p_{[\delta _q|\ell , u|w]})(F_q)$. By Lemma 2, we get $\delta _q< \delta < \delta _{q+1}$. However, by Lemma 2, we know that $\delta =w(s)$ for some $s\in S$, contradicting the definition of $\delta _{q+1}$. $\square$

**Fig. 1:**
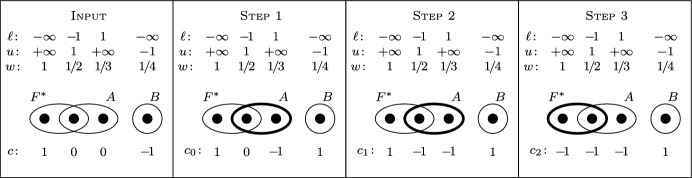
An illustration of Algorithm 1, where ${\mathcal {F}}=\{F^*,A,B\}$, the top three rows show the values of $\ell$, *u*, and *w*, respectively, and the costs *c* are presented at the bottom (Input). At each step, the set found by the oracle ${\mathcal {O}}$ is shown by a thick line

Note that Algorithm 1 runs in strongly polynomial time when we assume that ${\mathcal {O}}$ can be realized by a strongly polynomial-time algorithm. An illustration of the algorithm is given on Fig. 1.

### Binary search algorithm

With the help of Lemma 2, one can refine the search for $\delta$ by using a binary search technique for the sorted set $\{w(s)\}_{s\in S}$; this approach decreases the number of oracle calls from *O*(*n*) to $O(\log n)$. The algorithm is stated as Algorithm 2.

**Algorithm 2:**
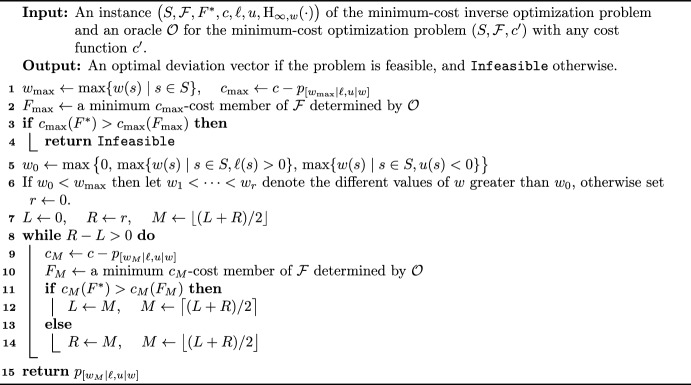
Binary search algorithm for the constrained minimum-cost inverse optimization problem under the weighted bottleneck Hamming distance objective

Let us show the correctness and the running time of the algorithm.

#### Theorem 5

Algorithm 2 determines an optimal deviation vector, if exists, for the minimum-cost inverse optimization problem $\big ( S, {\mathcal {F}}, F^*, c, \ell , u, \textrm{H}_{\infty , w}(\cdot ) \big )$ using $O(\log n)$ calls to the oracle ${\mathcal {O}}$.

#### Proof

We discuss the time complexity and the correctness of the algorithm separately.

*Time complexity.* The algorithm performs a binary search on the different values of *w*, which terminates after at most $\log n + 1$ iterations.

*Correctness.* By the above, the algorithm terminates after a finite number of iterations. Observe that if the algorithm returns Infeasible, then it correctly recognizes the problem to be infeasible by Lemma 3. Otherwise, feasibility follows by the choice of $w_0$ and Lemma 3, while optimality follows by Lemma 2. $\square$

## Weighted $\varvec{\ell _\infty }$-norm objective

Next we consider the weighted $\ell _{\infty }$-norm objective. Similarly to the case of the weighted bottleneck Hamming distance, we first prove that there exists an optimal deviation vector of a special form in Sect. 3.1. We characterize the feasibility of the problem in Sect. 3.2. Then we present an algorithm for the constrained setting in Sect. 3.3. Finally, we give a min-max characterization of the weighted $\ell _{\infty }$-norm of an optimal deviation vector in the unconstrained setting in Sect. 3.4.

### Optimal deviation vectors

Consider the constrained minimum-cost inverse optimization problem under the weighted $\ell _{\infty }$-norm objective $( S, {\mathcal {F}}, F^*, c, \ell , u, \Vert \cdot \Vert _{\infty ,w})$, where $w\in {\mathbb {R}}^S_+$ are positive weights. For any $\delta \ge 0$, let $p_{[\delta |\ell , u|w]}:S\rightarrow {\mathbb {R}}$ be defined as

$$\begin{aligned} p_{[\delta |\ell , u|w]}(s):={\left\{ \begin{array}{ll} \ell (s) & \text {if }s \in F^*\text { and }\delta /w(s)< \ell (s), \\ \delta /w(s) & \text {if }s \in F^*\text { and }\ell (s) \le \delta /w(s) \le u(s), \\ u(s) & \text {if }s \in F^*\text { and }u(s)< \delta /w(s), \\ \ell (s) & \text {if }s \in S\setminus F^*\text { and }- \delta /w(s)< \ell (s), \\ - \delta /w(s) & \text {if }s \in S\setminus F^*\text { and }\ell (s) \le - \delta /w(s) \le u(s), \\ u(s) & \text {if }s \in S\setminus F^*\text { and }u(s) < - \delta /w(s). \end{array}\right. } \end{aligned}$$

We simply write $p_{[\delta ||w]}$ when $\ell \equiv -\infty$ and $u\equiv +\infty$. The following technical lemma shows that there exists an optimal deviation vector of special form.

#### Lemma 6

Let $\left( S, {\mathcal {F}}, F^*, c, \ell , u, \Vert \cdot \Vert _{\infty , w} \right)$ be a feasible minimum-cost inverse optimization problem and let $p_{{\textsc {opt}}}$ be an optimal deviation vector. Then $p_{[\delta |\ell , u|w]}$ is also an optimal deviation vector, where $\delta :=\max \left\{ w(s) \cdot | p_{{\textsc {opt}}}(s) | \bigm | s \in S \right\}$.

#### Proof

The lower and upper bounds $\ell \le p_{[\delta |\ell , u|w]} \le u$ hold by definition, hence (b) is satisfied.

Now we show that (a) holds. The assumption $\ell \le p_{{\textsc {opt}}} \le u$ and the definition of $\delta$ imply that $-\delta /w(s) \le p_{{\textsc {opt}}}(s) \le u(s)$ and $\ell (s) \le p_{{\textsc {opt}}}(s) \le \delta /w(s)$ hold for every $s \in S$. Let $F \in {\mathcal {F}}$ be an arbitrary solution. Then

$$\begin{aligned}&(c - p_{[\delta |\ell , u|w]})(F^*) - (c - p_{[\delta |\ell , u|w]})(F)\\&\quad = \left( c(F^*) - \sum _{s \in F^*} p_{[\delta |\ell , u|w]}(s) \right) - \left( c(F) - \sum _{s \in F} p_{[\delta |\ell , u|w]}(s) \right) \\&\quad = c(F^*) - c(F) - \sum _{s \in F^*\setminus F} p_{[\delta |\ell , u|w]}(s) + \sum _{s \in F\setminus F^*} p_{[\delta |\ell , u|w]}(s) \\&\quad = c(F^*) - c(F) - \sum _{{\begin{array}{c} s \in F^*\setminus F \\ \delta /w(s)\ge \ell (s)\\ \delta /w(s) \le u(s) \end{array}}} \frac{\delta }{w(s)} - \sum _{{\begin{array}{c} s \in F^*\setminus F \\ u(s)< \delta /w(s) \end{array}}} u(s) + \sum _{{\begin{array}{c} s \in F\setminus F^* \\ - \delta /w(s) < \ell (s) \end{array}}} \ell (s) \\&\qquad + \sum _{{\begin{array}{c} s \in F\setminus F^* \\ -\delta /w(s)\ge \ell (s)\\ - \delta /w(s) \le u(s) \end{array}}} \left( - \, \frac{\delta }{w(s)} \right) \\&\quad \le c(F^*) - c(F) - \sum _{s \in F^*\setminus F} p_{{\textsc {opt}}}(s) + \sum _{s \in F\setminus F^*} p_{{\textsc {opt}}}(s) \\&\quad = (c-p_{{\textsc {opt}}})(F^*) - (c-p_{{\textsc {opt}}})(F) \\&\quad \le 0, \end{aligned}$$

where the last inequality holds by the feasibility of $p_{{\textsc {opt}}}$.

Finally, to see that (c) holds for $p_{[\delta |\ell , u|w]}$, recall that by the definition of $\delta$, we have $\ell (s) \le \delta /w(s)$ and $-\delta /w(s) \le u(s)$ for each $s\in S$, thus $\Vert p_{[\delta |\ell , u|w]} \Vert _{\infty , w} \le \delta = \Vert p_{{\textsc {opt}}} \Vert _{\infty , w}$. This concludes the proof of the lemma. $\square$

By Lemma 6, it suffices to look for an optimal deviation vector among vectors of special form. Furthermore, we get the following useful property of deviation vectors of such form.

#### Lemma 7

Let $\left( S, {\mathcal {F}}, F^*, c, \ell , u, \Vert \cdot \Vert _{\infty , w} \right)$ be a feasible minimum-cost inverse optimization problem and let $\delta \ge 0$ be such that $p_{[\delta |\ell , u|w]}$ is a feasible deviation vector. Then for any $\delta ' \ge \delta$, the deviation vector $p_{[\delta '|\ell , u|w]}$ is also feasible.

#### Proof

Let $F \in {\mathcal {F}}$ be an arbitrary solution. Then

$$\begin{aligned}&(c - p_{[\delta '|\ell , u|w]})(F^*) - (c - p_{[\delta '|\ell , u|w]})(F)\\&\quad = c(F^*) - c(F) - \sum _{{\begin{array}{c} s \in F^*\setminus F \\ \delta '/w(s)< \ell (s) \end{array}}} \ell (s) - \sum _{{\begin{array}{c} s \in F^*\setminus F \\ \delta '/w(s)\ge \ell (s)\\ \delta '/w(s) \le u(s) \end{array}}} \frac{\delta '}{w(s)} - \sum _{{\begin{array}{c} s \in F^*\setminus F \\ u(s)< \delta '/w(s) \end{array}}} u(s) \\&\quad \quad + \sum _{{\begin{array}{c} s \in F\setminus F^* \\ - \delta '/w(s)< \ell (s) \end{array}}} \ell (s) + \sum _{{\begin{array}{c} s \in F\setminus F^* \\ -\delta '/w(s)\ge \ell (s)\\ - \delta '/w(s) \le u(s) \end{array}}} \left( - \, \frac{\delta '}{w(s)} \right) + \sum _{{\begin{array}{c} s \in F\setminus F^* \\ u(s)< - \delta '/w(s) \end{array}}} \ell (s) \\&\quad \le c(F^*) - c(F) - \sum _{{\begin{array}{c} s \in F^*\setminus F \\ \delta /w(s)< \ell (s) \end{array}}} \ell (s) - \sum _{{\begin{array}{c} s \in F^*\setminus F \\ \delta /w(s)\ge \ell (s)\\ \delta '/w(s) \le u(s) \end{array}}} \frac{\delta }{w(s)} - \sum _{{\begin{array}{c} s \in F^*\setminus F \\ u(s)< \delta /w(s) \end{array}}} u(s) \\&\quad \quad + \sum _{{\begin{array}{c} s \in F\setminus F^* \\ - \delta /w(s)< \ell (s) \end{array}}} \ell (s) + \sum _{{\begin{array}{c} s \in F\setminus F^* \\ -\delta /w(s)\ge \ell (s)\\ - \delta /w(s) \le u(s) \end{array}}} \left( - \frac{\delta }{w(s)} \right) + \sum _{{\begin{array}{c} s \in F\setminus F^* \\ u(s) < - \delta /w(s) \end{array}}} \ell (s) \\&\quad = (c - p_{[\delta |\ell , u|w]})(F^*) - (c - p_{[\delta |\ell , u|w]})(F)\\&\quad \le 0, \end{aligned}$$

concluding the proof of the lemma. $\square$

### Characterizing feasibility

We give a necessary and sufficient condition for the feasibility of the minimum-cost inverse optimization problem $\left( S, {\mathcal {F}}, F^*, c, \ell , u, \Vert \cdot \Vert _{\infty , w} \right)$.

#### Lemma 8

Let $\left( S, {\mathcal {F}}, F^*, c, \ell , u, \Vert \cdot \Vert _{\infty , w} \right)$ be a minimum-cost inverse optimization problem. For any $F\in {\mathcal {F}}$, definewhich contradicts the definition of

$$\begin{aligned} A_1&:=\big \{ s \in F^* \setminus F \bigm | u(s) = +\infty \big \}, \\ A_2&:=\big \{ s \in F \setminus F^* \bigm | \ell (s) = -\infty \big \}, \end{aligned}$$

and

$$\begin{aligned} W(F):={\left\{ \begin{array}{ll} \displaystyle \frac{1}{\displaystyle \sum _{s \in A_1} \frac{1}{w(s)} + \sum _{s \in A_2} \frac{1}{w(s)}} & \text {if }A_1 \cup A_2 \ne \emptyset , \\ \,0 & \text {otherwise}. \end{array}\right. } \end{aligned}$$

Let

$$\begin{aligned} m_1 & :=\max \left\{ w(s) \cdot | u(s) | \bigm | s \in F^*,\, u(s) \ne + \infty \right\} ,\\ m_2 & :=\max \left\{ w(s) \cdot | \ell (s) | \bigm | s \in S\setminus F^*,\, \ell (s) \ne - \infty \right\} ,\\ m_3 & :=\max _{F \in {\mathcal {F}}} \left( W(F) \cdot \left( c(F^*) - c(F) - \sum _{\begin{array}{c} s \in F^*\setminus F \\ u(s) \ne + \infty \end{array}} u(s) + \sum _{\begin{array}{c} s \in F\setminus F^* \\ \ell (s) \ne - \infty \end{array}} \ell (s) \right) \right) . \end{aligned}$$

Then, the problem is feasible if and only if $p_{[m|\ell , u|w]}$ is a feasible deviation vector for

$$\begin{aligned} m:=\max \{0,m_1,m_2,m_3\}. \end{aligned}$$

#### Proof

Clearly, if $p_{[m|\ell , u|w]}$ is feasible, then so is the problem.

To see the other direction, suppose to the contrary that $p_{[m|\ell , u|w]}$ is not feasible, but there exists a feasible deviation vector *p*. Then there exists $F \in {\mathcal {F}}$ such that

$$\begin{aligned} 0 & < (c - p_{[m|\ell , u|w]})(F^*) - (c - p_{[m|\ell , u|w]})(F) \\ & = c(F^*) - c(F) - \sum _{\begin{array}{c} s \in F^*\setminus F \\ u(s) \ne + \infty \end{array}} u(s) - \sum _{\begin{array}{c} s \in F^*\setminus F \\ u(s) = + \infty \end{array}} \frac{m}{w(s)} + \sum _{\begin{array}{c} s \in F\setminus F^* \\ \ell (s) \ne - \infty \end{array}} \ell (s) + \sum _{\begin{array}{c} s \in F\setminus F^* \\ \ell (s) = - \infty \end{array}} \left( - \, \frac{m}{w(s)} \right) \\ & = c(F^*) - c(F) - \sum _{\begin{array}{c} s \in F^*\setminus F \\ u(s) \ne + \infty \end{array}} u(s) + \sum _{\begin{array}{c} s \in F\setminus F^* \\ \ell (s) \ne - \infty \end{array}} \ell (s) - m \left( \sum _{\begin{array}{c} s \in F^*\setminus F \\ u(s) = + \infty \end{array}} \frac{1}{w(s)} + \sum _{\begin{array}{c} s \in F\setminus F^* \\ \ell (s) = - \infty \end{array}} \frac{1}{w(s)} \right) . \end{aligned}$$

If $\{ s \in F^*{\setminus } F \mid u(s) = + \infty \} \cup \{ s \in F{\setminus } F^* \mid \ell (s) = - \infty \} = \emptyset$, then we obtain

$$\begin{aligned} 0 & < c(F^*) - c(F) - \sum _{\begin{array}{c} s \in F^*\setminus F \\ u(s) \ne + \infty \end{array}} u(s) + \sum _{\begin{array}{c} s \in F\setminus F^* \\ \ell (s) \ne - \infty \end{array}} \ell (s) - m \cdot 0 \\ & \le c(F^*) - c(F) - \sum _{s \in F^*\setminus F} p(s) + \sum _{s \in F\setminus F^*} p(s) \\ & = \big ( c(F^*) - p(F^*) \big ) - \big ( c(F) - p(F) \big )\\ & \le 0, \end{aligned}$$

where the last inequality holds since *p* is feasible, leading to a contradiction.

If ${\left\{ s \in F^*{\setminus } F \mid u(s) = + \infty \right\} } \cup \left\{ s \in F{\setminus } F^* \mid \ell (s) = - \infty \right\} \ne \emptyset$, then we obtain

$$\begin{aligned} 0 < c(F^*) - c(F) - \sum _{\begin{array}{c} s \in F^*\setminus F \\ u(s) \ne + \infty \end{array}} u(s) + \sum _{\begin{array}{c} s \in F\setminus F^* \\ \ell (s) \ne - \infty \end{array}} \ell (s) \, - \frac{m}{W(F)}, \end{aligned}$$

which contradicts the definition of *m*. $\square$

### Algorithm

We turn to the description of the algorithm and its analysis, which is similar to that of Algorithm 1. The algorithm is presented as Algorithm 3.

**Algorithm 3:**
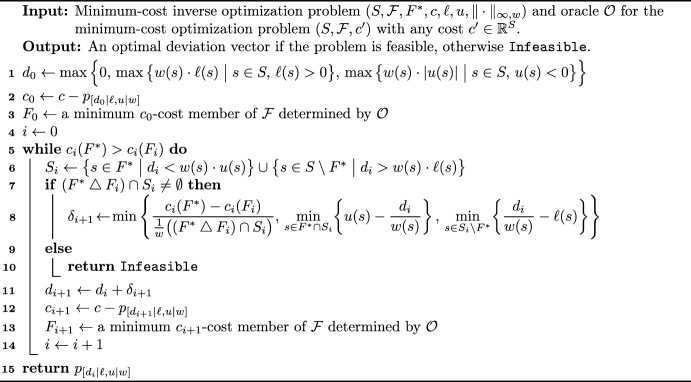
Algorithm for the constrained minimum-cost inverse optimization problem under the weighted $\ell _{\infty }$-norm objective

For proving the correctness and the running time of the algorithm, we need the following lemmas.

#### Lemma 9

If $F^*$ is not a minimum $c_i$-cost member of ${\mathcal {F}}$ for some *i*, then either $\delta _{i+1} > 0$ and $S_{i+1}\subseteq S_i$, or Algorithm 3 declares the problem to be infeasible.

#### Proof

The statement follows from the definition of $\delta _{i+1}$ and from Lemma 9. $\square$

#### Lemma 10

If $F^*$ is not a minimum $c_i$-cost member of ${\mathcal {F}}$ for some *i*, then $c_{i+1}(F^*) = c_{i+1}(F_i)$, or $S_{i+1} \subsetneq S_i$, or Algorithm 3 declares the problem to be infeasible.

#### Proof

Let *i* be an index such that $F^*$ is not a minimum $c_i$-cost member of ${\mathcal {F}}$, and assume that Algorithm 3 does not declare the problem to be infeasible in the *i*th step. Then $S_{i+1} \subseteq S_i$ holds by Lemma 9. If $S_{i+1} \subsetneq S_i$, then we are done, hence consider the case $S_{i+1} = S_i$. Then

$$\begin{aligned} \delta _{i+1} = \frac{c_i(F^*) - c_i(F_i)}{\textstyle \frac{1}{w} \big ( (F^* \mathbin {\triangle } F_i) \cap S_i \big )}, \end{aligned}$$

hence we get

$$\begin{aligned} c_{i+1}(F^*) - c_{i+1}(F_i) = c_i(F^*) - c_i(F_i) - \sum _{s \in (F^* \mathbin {\triangle } F_i) \cap S_i} \frac{\delta _{i+1}}{w(s)} = 0, \end{aligned}$$

concluding the proof of the lemma. $\square$

#### Lemma 11

If $F^*$ is not a minimum $c_i$-cost member of ${\mathcal {F}}$ for some *i*, then

$$\begin{aligned} \tfrac{1}{w} \big ( (F_i\setminus F^*) \cap S_i \big ) - \tfrac{1}{w} \big ( (F_i \cap F^*) \cap S_i \big ) > \tfrac{1}{w} \big ( (F_{i+1}\setminus F^*) \cap S_{i+1} \big ) - \tfrac{1}{w} \big ( (F_{i+1} \cap F^*) \cap S_{i+1} \big ) \end{aligned}$$

or $S_{i+1} \subsetneq S_i$, or Algorithm 3 declares the problem to be infeasible.

#### Proof

Let *i* be an index such that $F^*$ is not a minimum $c_i$-cost member of ${\mathcal {F}}$, and assume that Algorithm 3 does not declare the problem to be infeasible in the *i*-th step and that $S_{i+1}=S_i$. Then $c_{i+1}(F^*)=c_{i+1}(F_i)$ holds by Lemma 10. Thus we get

$$\begin{aligned} 0 & < c_{i+1}(F^*) - c_{i+1}(F_{i+1})\\ & = c_{i+1}(F_i) - c_{i+1}(F_{i+1}) \\ & = \left( c_i(F_i) - \sum _{{s \in (F_i \cap F^*) \cap S_i}} \frac{\delta _{i+1}}{w(s)} + \sum _{{s \in (F_i\setminus F^*) \cap S_i}} \frac{\delta _{i+1}}{w(s)} \right) \\ & ~~- \left( c_i(F_{i+1}) - \sum _{{s \in (F_{i+1} \cap F^*) \cap S_i}} \frac{\delta _{i+1}}{w(s)} + \sum _{{s \in (F_{i+1}\setminus F^*) \cap S_i}} \frac{\delta _{i+1}}{w(s)} \right) \\ & = c_i(F_i) - c_i(F_{i+1}) + \delta _{i+1} \cdot \Big [ \tfrac{1}{w} \big ( (F_i\setminus F^*) \cap S_i \big ) - \tfrac{1}{w} \big ( (F_i \cap F^*) \cap S_i \big ) \Big ]\\ & ~~~~- \delta _{i+1} \cdot \Big [ \tfrac{1}{w} \big ( (F_{i+1}\setminus F^*) \cap S_{i+1} \big ) - \tfrac{1}{w} \big ( (F_{i+1} \cap F^*) \cap S_{i+1} \big ) \Big ]. \end{aligned}$$

Since $\delta _{i+1}>0$ by Lemma 9 and $c_i(F_i)-c_i(F_{i+1})\le 0$ by the optimality of $F_i$ with respect to $c_i$, the statement follows. $\square$

With the help of Lemmas 9–11, we are ready to prove the main result of this section.

#### Theorem 12

Algorithm 3 determines an optimal deviation vector, if exists, for the minimum-cost inverse optimization problem $(S,{\mathcal {F}}, F^*, c, \ell , u, \Vert \cdot \Vert _{\infty , w})$ using $O(n\cdot \Vert w\Vert _{\text {-}1})$ calls to the ${\mathcal {O}}$.

#### Proof

We discuss the time complexity and the correctness of the algorithm separately.

*Time complexity.* Recall that $w\in {\mathbb {R}}^S_+$ is scaled so that $\frac{1}{w}(X)$ is an integer for each $X\subseteq S$. Between two iterations of the while loop, the size of the set $S_i$ or the value of $\frac{1}{w} \big ( (F_i{\setminus } F^*) \cap S_i \big ) - \frac{1}{w} \big ( (F_i \cap F^*) \cap S_i \big )$ strictly decreases by Lemma 11. The size of $S_i$ can decrease at most *n* times. Between two iterations where the size of $S_i$ decreases, the value of $\frac{1}{w} \big ( (F_i{\setminus } F^*) \cap S_i \big ) - \frac{1}{w} \big ( (F_i \cap F^*) \cap S_i \big )$ can decrease at most $2\Vert w\Vert _{\text {-}1}$ times. Hence the total number of iterations is $O(n\cdot \Vert w\Vert _{\text {-}1})$.

*Correctness.* By the above, the procedure terminates after a finite number of iterations. First, we show that if the the algorithm returns Infeasible, then it correctly recognizes the problem to be infeasible. To see this, assume that the algorithm terminated in the *i*th step and declared the problem to be infeasible. Then $(F^* \mathbin {\triangle } F_i) \cap S_i = \emptyset$, so by the definitions of $S_i$ and *m* as in Lemma 8, we obtain

$$\begin{aligned}&(c - p_{[m|\ell ,u|w]})(F^*) - (c - p_{[m|\ell ,u|w]})(F_i) \\&\quad = c(F^*) - c(F_i) - \sum _{\begin{array}{c} s \in F^*\setminus F_i \\ u(s) \ne + \infty \end{array}} u(s) - \sum _{\begin{array}{c} s \in F^*\setminus F \\ u(s) = + \infty \end{array}} \frac{m}{w(s)} + \sum _{\begin{array}{c} s \in F\setminus F^* \\ \ell (s) \ne - \infty \end{array}} \ell (s)\\&\qquad + \sum _{\begin{array}{c} s \in F\setminus F^* \\ \ell (s) = - \infty \end{array}} \left( - \, \frac{m}{w(s)} \right) \\&\quad = c(F) - c(F_i) - \sum _{s \in F^*\setminus F_i} u(s) + \sum _{s \in F_i\setminus F^*} \ell (s)\\&\quad = (c - p_{[d_i|\ell , u|w]})(F^*) - (c - p_{[d_i|\ell , u|w]})(F_i)\\&\quad ~~> 0, \end{aligned}$$

hence the problem is infeasible by Lemma 8.

Assume now that the algorithm terminates with returning a deviation vector whose feasibility follows from the fact that the while loop ended. If $F^*$ is a minimum $c_0$-cost member of ${\mathcal {F}}$, then we are clearly done. Otherwise, there exists an index *q* such that $F^*$ is a minimum $c_{q+1}$-cost member of ${\mathcal {F}}$. Suppose to the contrary that $p_{[d_{q+1}|\ell , u|w]}$ is not optimal. By Lemma 6, there exists $\delta <d_{q+1}$ such that the deviation vector $p_{[\delta |\ell , u|w]}$ is optimal. Thus, by Lemma 10, we get

$$\begin{aligned} 0 & \ge (c - p_{[\delta |\ell , u|w]})(F^*) - (c - p_{[\delta |\ell , u|w]})(F_q) \\ & = c(F^*) - c(F_q) - \sum _{{\begin{array}{c} s \in F^*\setminus F_q \\ \delta /w(s) \le u(s) \end{array}}} \frac{\delta }{w(s)} - \sum _{{\begin{array}{c} s \in F^*\setminus F_q \\ u(s)< \delta /w(s) \end{array}}} u(s) \\&\quad + \sum _{{\begin{array}{c} s \in F_q\setminus F^* \\ - \delta /w(s)< \ell (s) \end{array}}} \ell (s) + \sum _{{\begin{array}{c} s \in F_q\setminus F^* \\ \ell (s) \le - \delta /w(s) \end{array}}} \left( - \, \frac{\delta }{w(s)} \right) \\ & > c(F^*) - c(F_q) - \hspace{-0.2cm}\sum _{{\begin{array}{c} s \in F^*\setminus F_q \\ d_{q+1}/w(s) \le u(s) \end{array}}} \frac{d_{q+1}}{w(s)} - \hspace{-0.2cm}\sum _{{\begin{array}{c} s \in F^*\setminus F_q \\ u(s)< d_{q+1}/w(s) \end{array}}} u(s) + \hspace{-0.2cm}\sum _{{\begin{array}{c} s \in F_q\setminus F^* \\ - d_{q+1}/w(s) < \ell (s) \end{array}}} \ell (s) \\&\quad + \hspace{-0.2cm}\sum _{{\begin{array}{c} s \in F_q \setminus F^* \\ \ell (s) \le - d_{q+1}/w(s) \end{array}}} \left( - \, \frac{d_{q+1}}{w(s)} \right) \\ & = c_{q+1}(F^*) - c_{q+1}(F_q)\\ & = 0, \end{aligned}$$

a contradiction. This concludes the proof of the theorem. $\square$

**Fig. 2:**
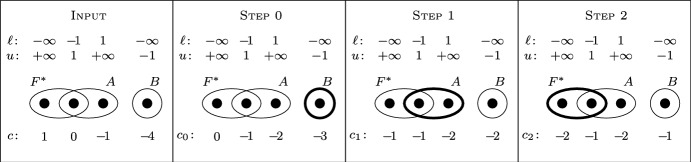
An illustration of Algorithm 3, where ${\mathcal {F}}=\{F^*,A,B\}$, the weights are identically 1, the top two rows show the values of $\ell$ and *u*, respectively, and the costs *c* are presented at the bottom (Input). At each step, the set found by the oracle ${\mathcal {O}}$ is shown by a thick line

Note that the Algorithm 3 runs in strongly polynomial time assuming that ${\mathcal {O}}$ can be realized by a strongly polynomial-time algorithm. An illustration of the algorithm is given on Fig. 2.

### Min-max theorem

With the help of Lemmas 6 and 7, we provide a min-max characterization for the weighted infinity norm of an optimal deviation vector in the unconstrained setting. The interpretation of this min-max result is the following. Each member $F \in {\mathcal {F}}$ requires some extent of modification in the costs. Clearly, the minimum extent of modification is at least the maximum of the extent of those requirements. However, by the min-max result, we do not need to make a larger modification than that. Therefore, given a member $F \in {\mathcal {F}}$ requiring the largest extent of modification $\delta$, we know that $p_{[\delta ||w]}$ is optimal. Note that Algorithm 3 traces such a member.

Recall that we use the notation $\frac{1}{w}(X):=\sum _{s \in X} \frac{1}{w(s)}$.

#### Theorem 13

Let $\big ( S, {\mathcal {F}}, F^*, c, -\infty , +\infty , \Vert \cdot \Vert _{\infty , w} \big )$ be a minimum-cost inverse optimization problem. Then

$$\begin{aligned} & \min \big \{\Vert p \Vert _{\infty , w} \bigm | p\text { is a feasible deviation vector} \big \} \\ & \quad = \max \left\{ 0, \, \max \left\{ \frac{c(F^*) - c(F)}{\frac{1}{w}(F^* \mathbin {\triangle } F)} \, \big | \, F \in {\mathcal {F}}, \, F \ne F^* \right\} \right\} . \end{aligned}$$

#### Proof

By Lemma 6, there exists $\delta \ge 0$ such that $p_{[\delta ||w]}$ is an optimal deviation vector for the problem $( S, {\mathcal {F}}, F^*, c, -\infty , +\infty , \Vert \cdot \Vert _{\infty , w})$.

For ease of discussion, let us define

$$\begin{aligned} d :=\max \left\{ \frac{c(F^*) - c(F)}{\frac{1}{w}(F^* \mathbin {\triangle } F)}\,\big |\, F \in {\mathcal {F}}, \, F \ne F^* \right\} . \end{aligned}$$

Our goal is to show that $\delta = \max \{ 0, d \}$.

If $F^*$ is a minimum *c*-cost member of ${\mathcal {F}}$, then we are clearly done. Otherwise, $\delta , d > 0$ holds, and it suffices to show $\delta = d$. Let $F \in {\mathcal {F}}$, $F \ne F^*$ be arbitrary. Since $p_{[\delta ||w]}$ is feasible, we obtain

$$\begin{aligned} 0 & \ge (c - p_{[\delta ||w]})(F^*) - (c - p_{[\delta ||w]})(F) \\ & = \left( c(F^*) - \sum _{s \in F^*} \frac{\delta }{w(s)} \right) - \left( c(F) - \sum _{s \in F \cap F^*} \frac{\delta }{w(s)} - \sum _{s \in F\setminus F^*} \left( - \, \frac{\delta }{w(s)} \right) \right) \\ & = c(F^*) - c(F) - \sum _{s \in F^* \mathbin {\triangle } F} \frac{\delta }{w(s)} \\ & = c(F^*) - c(F) - \delta \cdot \tfrac{1}{w}(F^* \mathbin {\triangle } F). \end{aligned}$$

This implies

$$\begin{aligned} \delta \ge \frac{c(F^*) - c(F)}{\frac{1}{w}(F^* \mathbin {\triangle } F)}, \end{aligned}$$

hence $\delta \ge d$. To prove $\delta \le d$, it is enough to show that $p_{[d||w]}$ is a feasible deviation vector. For any $F \in {\mathcal {F}}$, $F \ne F^*$, we have

$$\begin{aligned} (c - p_{[d||w]})(F^*) - (c - p_{[d||w]})(F) & = c(F^*) - c(F) - d \cdot \tfrac{1}{w}(F^* \mathbin {\triangle } F) \\ & \le c(F^*) - c(F) - \frac{c(F^*) - c(F)}{\frac{1}{w}(F^* \mathbin {\triangle } F)} \cdot \tfrac{1}{w}(F^* \mathbin {\triangle } F)\\ & = 0, \end{aligned}$$

which means that $p_{[d||w]}$ is indeed feasible. $\square$

#### Remark 14

Theorem 13 provides further insight into why the proposed framework is referred to as *Newton-type*. For simplicity, consider a minimum-cost inverse optimization problem of the form $( S, {\mathcal {F}}, F^*, c, -\infty , +\infty , \Vert \cdot \Vert _{\infty , w} )$ with uniform weights set to 1, and assume that the optimal solution is nonzero, meaning that the maximum in Theorem 13 is attained by the second term. The min-max result states that a deviation vector $p^*$ with $\delta ^* :=\Vert p^* \Vert _{\infty ,w}$ is optimal if and only if

$$\begin{aligned} \max \left\{ \frac{c(F^*) - c(F)}{|F^* \mathbin {\triangle } F|} - \delta ^* \, \big | \, F \in {\mathcal {F}}, \, F \ne F^* \right\} = 0. \end{aligned}$$

Thus, finding $\delta ^*$ is equivalent to locating a root of the function

$$\begin{aligned} f(\delta ) :=\max \left\{ \frac{c(F^*) - c(F)}{|F^* \mathbin {\triangle } F|} - \delta \, \big | \, F \in {\mathcal {F}}, \, F \ne F^* \right\} . \end{aligned}$$

Our algorithms, similar to Newton’s method, aim to find a root of this function: in the *i*-th iteration, $\delta _{i+1}$ is computed as the solution to the equation $\frac{c(F^*) - c(F_i)}{|F^* \mathbin {\triangle } F_i|} - \delta = 0$.

## Conclusions

In this paper, we considered general minimum-cost inverse optimization problems in the constrained setting, i.e. with lower and upper bounds on the coordinates of the deviation vector. We provided simple, purely combinatorial algorithms for the weighted bottleneck Hamming distance and $\ell _{\infty }$-norm objectives. The algorithms follow a scheme that resembles Newton’s algorithm. For the weighted bottleneck Hamming distance objective, the algorithm runs in strongly polynomial time, assuming that a strongly polynomial algorithm for the underlying optimization problem is available. We also exhibited a faster binary search algorithm for this objective. For the weighted $\ell _{\infty }$-norm objective, the algorithm runs in pseudo-polynomial time in general and in strongly polynomial time for unit weights, assuming that a strongly polynomial algorithm for the underlying optimization problem is available.

Despite the extensive literature on inverse optimization, only few results are known when the desired deviation vector *p* is required to be integral, see e.g. [1, 16, 17]. If $\ell$, *u* and *c* are integral vectors, then the deviation vector $p_{[\delta |\ell , u|w]}$ defined in the bottleneck Hamming distance case is integral independently from the choice of $\delta$, and therefore the fractional and integral optima coincide. For the unweighted $\ell _{\infty }$-norm objective, i.e. when in addition $w\equiv 1$ holds, the deviation vector $p_{[\delta |\ell , u|w]}$ might not be integral as we assign value $\delta$ to some of its coordinates. However, Lemmas 6 and 7 together imply that if $p_{[\delta |\ell , u|w]}$ is an optimal fractional deviation vector, then $p_{[\lceil \delta \rceil |\ell , u|w]}$ is an optimal integral deviation vector.

We close the paper by mentioning an open problem. Lemmas 1 and 6 prove the existence of optimal deviation vectors that have strong structural properties. However, these statements do not give any information on the complete set of optimal deviation vectors. Therefore, it remains an interesting question whether one can give a characterization of the set of all optimal deviation vectors.

## Data Availability

No new data were created or analysed in this study. Data sharing is not applicable to this article.
